# Photocatalytic Oxygen Evolution with Prussian Blue Coated ZnO Origami Core‐Shell Nanostructures

**DOI:** 10.1002/cphc.202400817

**Published:** 2025-01-29

**Authors:** Ruby Phul, Guobin Jia, Emir Utku Sekercileroglu, Yves Carstensen, Ratnadip De, Andrea Dellith, Jan Dellith, Jonathan Plentz, Ferdi Karadaş, Benjamin Dietzek‐Ivanšić

**Affiliations:** ^1^ Department of Chemistry Science Faculty Bilkent University Main Campus, Bilkent Çankaya, Ankara 06800 Türkiye; ^2^ Leibniz Institute of Photonic Technology (Leibniz-IPHT) Albert-Einstein-Str. 9 07745 Jena Germany; ^3^ Institute for Physical Chemistry and Abbe Center of Photonics Friedrich Schiller University Jena Helmholtzweg 4 07743 Jena Germany; ^4^ UNAM – National Nanotechnology Research Center Institute of Materials Science and Nanotechnology Bilkent University 06800 Ankara Türkiye

**Keywords:** Prussian blue, ZnO origami structure, Oxygen evolution reaction (OER), Core-shell heterojunction, Visible light photocatalyst

## Abstract

The design and development of particulate photocatalysts have been an attractive strategy to incorporate earth‐abundant metal ions to water splitting devices. Herein, we synthesized CoFe‐Prussian blue (PB) coated ZnO origami core‐shell nanostructures (PB@ZnO) with different massratios of PB components and investigated their photocatalytic water oxidation activities in the presence of an electron scavenger. Photocatalytic experiments reveal that the integration of PB on ZnO boosts the oxygen evolution rate by a factor of ~2.4 compared to bare ZnO origami. We ascribe this increased photocatalytic rate to an improved charge carrier separation and transfer due to the formation of heterojunction at the interface between PB and ZnO. Long‐term photocatalytic experiments indicate that the activity and stability of the catalyst was preserved up to 9 h. Our results indicate that the core‐shell PB@ZnO particles possess a proper band energy alignment for the photocatalytic water oxidation process.

## Graphical abstract

Prussian Blue and its derivative hold significant potential for photocatalytic oxygen evolution. IN this contribution the photocatalytic oxygen evolution reaction (OER) was characterized using a hybrid catalyst consisting of Prussian Blue coated ZnO nanostructures. The resulting heterojunction, which was characterized both structurally and functionally, facilitated charge carrier separation and transfer.

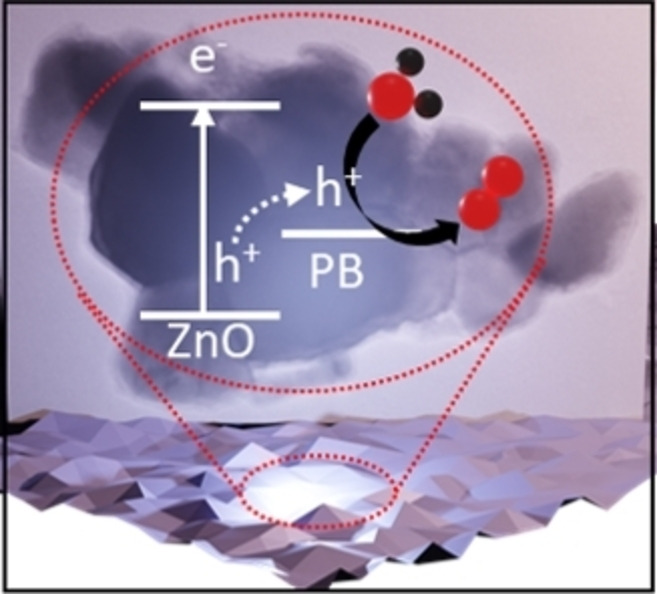

## Introduction

The oxygen evolution reaction (OER) is one of the half‐reactions of the overall water splitting process. It involves a four‐electron oxidative transformation, which is associated with a high reaction barrier,^[1]^ making it considerably more sluggish compared to the H_2_ evolution reaction (HER). Nonetheless, photocatalytic OER (as part of artificial photosynthetic water splitting) offers a sustainable method for producing O_2_ and H_2_ by harnessing sunlight thereby replacing fossil energy sources and enabling the conversion of solar energy into stored chemical energy. However, the development of efficient and stable photocatalysts is crucial for advancing the practical application of solar‐to‐hydrogen energy conversion technologies.

Zinc oxide (ZnO) stands out as a highly promising semiconductor material due to its abundance, eco‐friendly properties, high photosensitivity, and excellent chemical stability.^[2]^ Similar to TiO_2_, ZnO has a wide band gap (ca. 3.2 eV), restricting its intrinsic absorption to the ultraviolet (UV) region, in which only a small fraction (3–5 %) of solar light can be harvested. Furthermore, the fast recombination of photogenerated electron‐hole pairs in ZnO limits its overall photocatalytic efficiency.^[3]^ To overcome these limitations and to improve the photocatalytic efficiency of ZnO, various strategies have been employed, including metal doping,^[4]^ carbon or nitrogen doping,[^3^, ^5^] creation of heterostructure with other metal oxides,^[5]^ introducing oxygen vacancies,^[6]^ and deposition of co‐catalyst onto ZnO.^[7]^ Recently, ZnO origami was introduced, i. e. quasi 2D nanosheets, which were shown to exhibit promising activity for visible light induced photocatalytic degradation of methylene blue even at a wavelength of 505 nm, which greatly extends the energy harvesting range of the solar light.^[8]^ This remarkable visible light‐driven photocatalytic activity was attributed to the intrinsic defects in the ZnO origami.^[9]^ Briefly, the morphology of ZnO plays an important role for its defect‐related photocatalytic activity. Carriers can be generated by absorption of visible light via interband defect levels, and they migrate to the surface quickly due to the short distance to the surface, so that the recombination loss can be suppressed.

Prussian blue analogues (PBAs)^[10]^ are classical coordination polymers, which received significant interest in various fields in recent years, including photothermal conversion for controlled drug delivery applications,^[11]^ electrochromic,^[12]^ sodium ion batteries,^[13]^ photomagnetic properties,^[14]^ and environmental remediation, due to its unique properties. Its composition, typically A_a_M_b_[M’_c_(CN)₆], (A=alkali metal, M, M’=3d transition metal ions) offers an easy tuning of the identity of the metal ion as well as the M:M’ ratio, which controls the PBAs electronic and optical properties. We have previously reported that the photocatalytic performance of different semiconductors could be enhanced significantly when they are coupled with Co−Fe PBAs.[^15^, ^16^] Abundant redox active Co sites within the PBA framework make it a promising catalyst for water oxidation reaction.[^17^, ^18^] Activation of Co−Fe PBAs for water oxidation requires oxidation of Co^II^ to Co^III^. However, the lower absorption cross‐section of the required charge transfer (CT) transition (Co^II^ → Fe^III^ CT transitions are essential for photochemical activation of PBA) renders PBAs rather inefficient light harvesting units.^[19]^ In this context, an efficient approach to exploit the redox activity of PBAs towards water oxidation is to combine them with light harvesting metal oxide semiconductors to produce PBA/semiconductor hybrid heterostructures.[^20^, ^21^] This strategy not only circumvents the inefficient light harvesting ability of the PBA but also mitigates the fast charge recombination of metal oxide semiconductors and allows charge transfer across the PBA/semiconductor interface.^[22]^ For example, surface modification of a WO_3_/BiVO_4_ photoanodes by PBA layers led to suppressed recombination of photogenerated holes in BiVO_4_ resulting 20 times increase in incident photon to current conversion efficiency (IPCE).^[23]^ Similarly, Tang et al reported composite of PBA with core shell Fe_2_O_3_/Fe_2_TiO_5_ that showed one order of magnitude increased photocurrent in comparison to the pristine Fe_2_O_3_ nanowires.^[24]^ Our group studied the photocatalytic water oxidation performance of such hybrid semiconductors by coupling PBA with metal oxide semiconductors such as SrTiO_3_ and TiO_2_ that results in a 2 to 7‐fold increase in the photocatalytic yield as compared to the pristine metal oxides.[^15^, ^16^] These studies underscore the synergistic effects that can be achieved by combining PBA with metal oxide semiconductors.

Herein, we have prepared a PB@ZnO core‐shell structure as a photocatalyst for the OER process. We prepared a series of PB@ZnO assemblies with different mass ratios of PB:ZnO and performed photocatalytic activities. The origin of enhancement in the photocatalytic activity was elucidated with characterization studies.

## Results and Discussion

### Synthesis and Characterization

#### Chemicals

Zinc nitrate hexahydrate Zn(NO_3_)_2_ ⋅ 6H_2_O (≥99.0 %) was purchased from Sigma‐Aldrich and NH_4_OH (25 %) from Th. Geyer. The K_3_[Fe(CN)_6_] and Co(NO_3_)_2_ ⋅ 6H_2_O salts are purchased from Thermo Scientific with purities of ≥99.0 % and 99 %, respectively. All the chemicals were used as received without further purification.

#### Preparation of ZnO Origami

The Zn(OH)_2_ origami is prepared by an interfacial growth process developed recently. Following the previously reported protocol,^[8]^ a thin film of Zn(OH)_2_ is grown at the air/water interface by decomposition of zinc ammine hydroxide Zn(NH_3_)_x_(OH)_2_. The resultant Zn(OH)_2_ origami structures consist of 2D nanosheets, and ZnO nanoparticles can be obtained by annealing, so that the Zn(OH)_2_ converts to ZnO by dehydration. In this work, the annealing of the Zn(OH)_2_ origami has been done at 600 °C for 30 min.

#### Preparation of PB@ZnO Core‐Shell Structures

The synthesis of the PB@ZnO is performed by impregnation of the ZnO origami (annealed at 600 °C for 30 min) in K_3_[Fe(CN)_6_] and Co(NO_3_)_2_ solutions, alternately. 1 equivalent (eq.) of K_3_[Fe(CN)_6_] solution is added to the finely milled ZnO origami powders, and stirred for ~24 h at room temperature. Afterward, a cleaning procedure is performed to remove the residuals. The mixture is centrifuged at 6000 U/min for 5 min, and the supernatant is discarded, fresh high purity water (18.2 MΩ ⋅ cm) prepared by a Milli‐Q device is added and the sediment is stirred and sonicated. Then the centrifugation is done again at 6000 U/min for 5 min, and totally five times centrifugation is needed to clean the mixture. After five cycles of cleaning, a 1.5 eq. of Co(NO_3_)_2_ ⋅ 6H_2_O is added to the sediments and stirred for around 24 h, and the mixture is cleaned five times by the same procedure. The above described process is one cycle of the synthesis, and the obtained product is labeled as PB1@ZnO. By further cycles, PB2@ZnO and PB3@ZnO are obtained.

**Figure 1:**
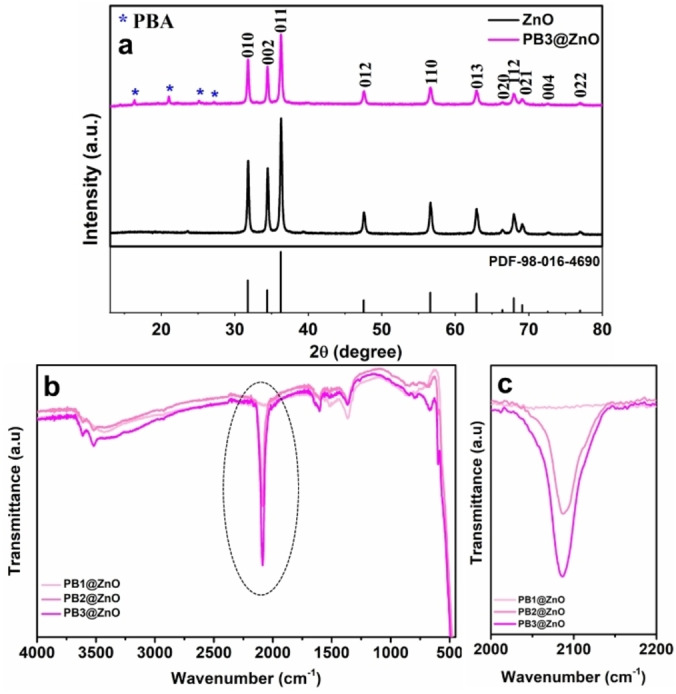
(a) XRD patterns of the ZnO origami and the PB3@ZnO core‐shell structures (the standard diffraction pattern for hexagonal ZnO is shown at the bottom), (b), and (c) FT‐IR spectra of different PB@ZnO structures, with their cyanide stretching modes.

**Figure 2:**
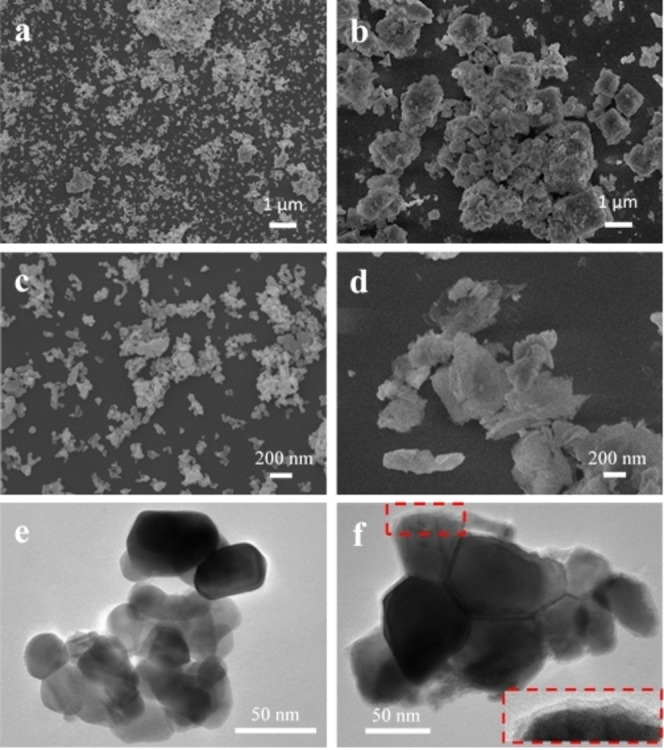
(a) and (b) The SEM images and (c, d) their corresponding highly magnified SEM images for ZnO origami annealed at 600 °C for 30 min and PB3@ZnO origami, respectively. (e) and (f) The TEM images of ZnO origami annealed at 600 °C and PB3@ZnO core‐shell structures (the inset in (f) is a magnified view of the area marked with a red rectangle).

The X‐ray diffraction (XRD) patterns of the resultant core‐shell structures are depicted in Figure 1a. The patterns can be indexed to the hexagonal wurtzite structure of ZnO, which matches well with the standard diffraction data of ZnO (PDF‐98‐016‐4690). The XRD patterns of all PB@ZnO assemblies are similar with only slight difference in the relative intensities of the peaks, indicating that the PB coating does not alter the crystal structure of the underlying ZnO. Additional low‐intensity peaks at 16.31°, 20.97°, 25.14°, and 27.18° in the diffraction pattern of PB3@ZnO confirm the presence of a thin coating of PB structure on the ZnO surface.^[24]^ The FT‐IR spectra of PB@ZnO origami structures are shown in Figures 1b and c. The strong band at 2085 cm^−1^ corresponds to the stretching vibration of CN^−^ groups in the PB structure. A weak band at ~602 cm^−1^ is attributed to Co−N and Fe−C coordination bonds.[^26^, ^27^] The bands at ~1610 cm^−1^ and 3620 cm^−1^ are assigned to the OH stretching and bending modes of water molecules present at the surface and the interstitial lattices of the PB structure.^[28]^ The surface morphology of the ZnO origami and the PB3@ZnO core‐shell structures were investigated by scanning electron microscopy (SEM). Figure 2a and the corresponding high magnification SEM image in Figure 2c show the morphology of the ZnO origami structure annealed at 600 °C for 30 min. The data reveal ZnO nanoparticles with sizes below ~50 nm alongside some large clusters of such small nanoparticles. The transmission electron microscopy (TEM) image in Figure 2e shows the structure of such cluster, which consists of smaller nanoparticles sticking together. On the contrary, the PB3@ZnO (Figure 2b and d) particles exhibit somewhat cubic morphology, confirming the formation of a PB layer over the ZnO structure.[^29^, ^30^] The TEM image of the PB3@ZnO (Figure 2f) shows a core‐shell structure with a shell thickness of 3.1±0.3 nm.

**Figure 3:**
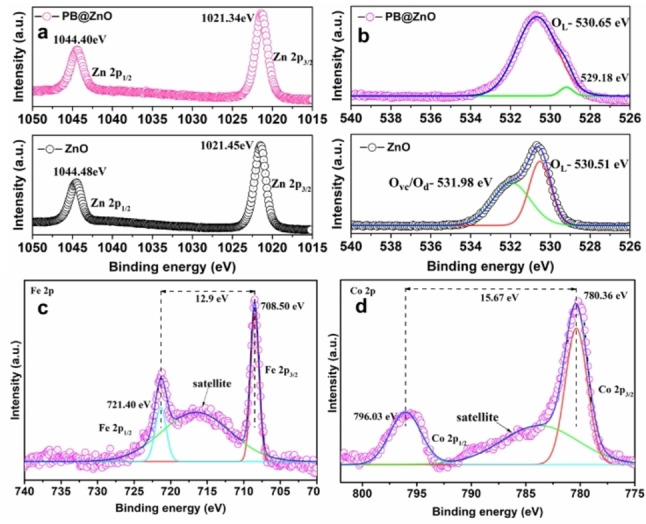
High‐resolution XPS spectra of (a) Zn 2p, (b) O 1s, (c) Fe 2p, and (d) Co 2p for ZnO and PB3@ZnO.

Analysis by X‐ray photoelectron spectroscopy (XPS; see Figure 3) focuses on the high‐resolution XPS spectra of Zn 2p, O 1s, Fe 2p, and Co 2p both for ZnO and PB3@ZnO samples. The Zn 2p spectra exhibit two distinctive peaks at binding energies of ca. 1044.5 and 1021.4 eV, corresponding to the Zn 2p_1/2_ and Zn 2p_3/2_ orbitals, respectively (Figure 3a). The spin orbit splitting energy between Zn 2p_1/2_, and Zn 2p_3/2_ orbitals was calculated to be 23.1 eV, confirming the presence of Zn^II^ confined in the ZnO structure.^[31]^ The typical O 1s spectra of ZnO (Figure 3b) was fitted with two Gaussian peaks at the binding energies around 530.5 and 531.9 eV. The peak at 530.5 eV is attributed to the lattice oxygen (O_L_), while the peak at 531.9 eV could be assigned to the oxygen vacancy (O_V_) and/or oxygen defect (O_D_).^[32]^ Interestingly, the O 1s spectra of PB3@ZnO showed only one peak, which corresponds to O_L_. Under otherwise identical conditions the O_V_ and/or O_D_ peaks were undetectable due to the formation of a thick layer of PB structure on ZnO. Furthermore, the Fe 2p spectra were deconvoluted into Fe 2p3/2 (708.5 eV) and Fe 2p1/2 (721.4 eV) peaks, each corresponding to the Fe^II^ state (Figure 3c). Two peaks at 780.3 and 796.0 eV were fitted to the high‐resolution Co 2p spectra, representing 2p3/2 and 2p1/2 states of both Co^II^ and Co^III^ (Figure 3d).^[33]^ A satellite peak was observed between the 3/2 and 1/2 states of both Fe 2p and Co 2p spectra. The spin‐orbit separation energy for Fe 2p and Co 2p was calculated to be 12.9 eV and 15.7 eV, respectively, demonstrating that Fe is present in the Fe^II^ state, whereas Co is in both Co^II^ and Co^III^ states. These results are also in agreement with cyanide stretches observed in Infrared studies.

#### Photocatalytic Studies

**Figure 4:**
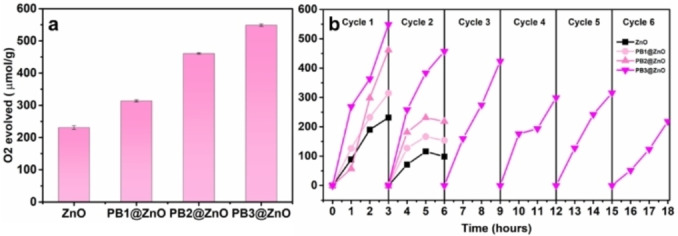
Photocatalytic oxygen evolution profiles of ZnO, PB1@ZnO, PB2@ZnO, and PB3@ZnO in 0.5 M persulfate solution under UV‐vis irradiation (300 W Xe lamp) during (a) a 3 h experiment and (b) six cycles totaling 18 h. The water oxidation performance of persulfate solution was also assessed and used as blank.

The photocatalytic activity of ZnO, PB1@ZnO, PB2@ZnO, and PB3@ZnO towards oxygen evolution was evaluated in the presence of 5 mM Na_2_S_2_O_8_ as a sacrificial oxidant under simulated solar irradiation. During the photocatalytic tests the samples were irradiated for 3 h per catalytic cycle (see Figure 4a). The oxygen evolution activity of persulfate was used as blank, and the activity was subtracted from the data obtained for the ZnO and PB@ZnO samples. Under these conditions the ZnO origami evolved 231 μmol/g of oxygen in 3 h, while increasing the thickness of the PB coating led to a remarkable enhancement enhancing of the activity. The PB3@ZnO sample showed ~75 % higher activity (549 μmol/g) than PB1@ZnO sample (314 μmol/g), and 238 % enhanced activity compared to ZnO during a 3 h photocatalytic experiment. Furthermore, the stability of the catalyst (PB3@ZnO) was tested through six consecutive cycles of the photocatalytic oxygen evolution experiment. After each cycle, the suspension was centrifuged and the supernatant was discarded, followed by washing with deionized water, and discarding the supernatant after a further centrifugation step, and then fresh 5 mM Na_2_S_2_O_8_ solution was added. Figure 4b shows that the activity and stability of the sample were almost retained up to 9 h of the photocatalytic experiment. However, a notable decrease in activity was observed from 9 h onwards, which could be partly attributed to the loss of catalyst during the washing process or the relatively low stability of the photocatalyst. The higher activity of PB3@ZnO sample suggests an enhanced ability for charge separation as compared to bare ZnO.

#### Band‐Energy Alignment

**Figure 5:**
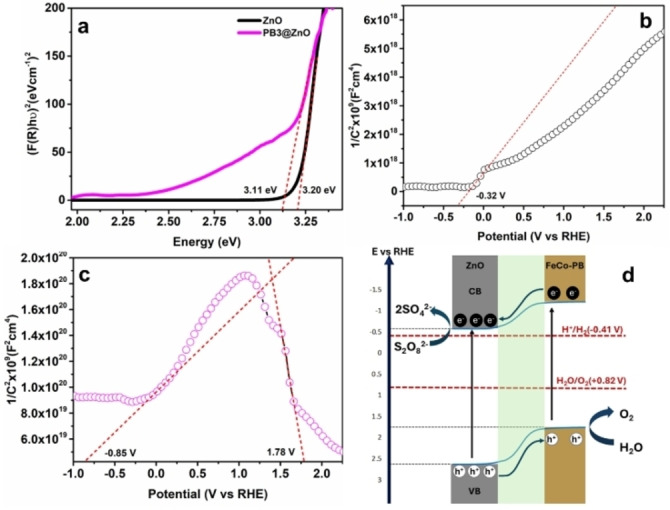
(a) Tauc plot for direct band gap determination derived from UV‐vis DRS, and (b) and (c) Mott–Schottky plots at 500 hz for ZnO and PB3@ZnO, respectively, in 0.1 M PBS electrolyte (pH 7) at a scan rate of 50 mV/s. The red lines represent the flat band potential values. (d) Schematic illustration of PB@ZnO‐ p‐n junction and photogenerated electrons & holes transfer mechanism for the photocatalytic water oxidation process.

To elucidate the origin of enhanced activity of PB3@ZnO, the band alignment in the heterostructure was studied. UV‐vis diffuse reflectance spectroscopy (UV‐vis DRS) measurement yielded the bandgap of the semiconductor using Tauc plot. Figure 5a reveals a direct bandgap of ZnO and PB3@ZnO of 3.20 and 3.11 eV, respectively. The electrochemical measurements (cyclic voltammetry and Mott‐Schottky) were carried out to determine the valence band (E_v_), conduction band (E_c_) of ZnO, and the energy levels of the PB structure. The Mott‐Schottky plots of ZnO and PB3@ZnO electrodes were acquired to determine the flat band potential (V_FB_) at 500 Hz (Figure 5b and c). The positive slope for ZnO reflects the n‐type behavior of the semiconductor, and the flat band potential was obtained as −0.32 V (see Figure 5b), which is consistent with the literature.[^34^, ^35^] On the contrary, the p‐type nature of PB structure is verified by the Mottt‐Schottky analysis of the PB3@ZnO core‐shell structure, which agrees very well with the literature.^[36]^ The inverted V‐shaped Mott‐Schottky plot for the core‐shell structure indicates the formation of a p‐n junction,[^15^, ^37^] which could be the origin of enhanced photocatalytic water oxidation activity of the core‐shell structure. The p‐n junction creates a strong electric field in the space charge region and reduces the charge transfer resistance at the interface, thereby enhancing the charge carrier dynamics.^[38]^ Furthermore, the cyclic voltammetry was performed to elucidate the HOMO level of PB structure, which is obtained as ~1.85 V vs RHE (see Figure S1). As the valence band of ZnO lies at a more positive potential with respect to that of PB structure, the PB3@ZnO core‐shell structure displays a proper band energy alignment (see Figure 5d) for the prompt hole transfer from the ZnO to the PB structure during the photocatalytic water oxidation process.

## Conclusions

FeCo Prussian blue coated ZnO core‐shell structures (PB@ZnO) were prepared with different PB:ZnO mass ratios. The photocatalytic water oxidation activity of PB@ZnO assemblies and bare ZnO were performed in the presence of an electron scavenger to evaluate the effect of PB coating on the catalytic activity. Photocatalytic experiments reveal that all PB@ZnO samples exhibit a higher activity than bare ZnO origami and activity increases as the mass ratio of PB increases in the hybrid assembly. The one with the highest mass ratio, i. e. PB3@ZnO, exhibited the highest activity of 549 μmol/g while it is only 231 μmol/g for bare ZnO during a 3 h experiment. Furthermore, PB3@ZnO exhibits a higher activity and stability during a six‐cycle photocatalytic experiment compared to other PB@ZnO assemblies and bare ZnO. Comprehensive characterization studies show that PB@ZnO exhibits a p‐n type heterojunction and a proper band energy alignment for efficient charge transfer and separation, unveiling the origin of enhanced activity observed in PB@ZnO samples. These findings underline the importance of choosing proper components for particulate suspensions in photocatalysis.

## Conflict of Interests

The authors declare no conflict of interest.

1

## Supporting information

As a service to our authors and readers, this journal provides supporting information supplied by the authors. Such materials are peer reviewed and may be re‐organized for online delivery, but are not copy‐edited or typeset. Technical support issues arising from supporting information (other than missing files) should be addressed to the authors.

Supporting Information

## Data Availability

The data that support the findings of this study are available from the corresponding author upon reasonable request.
