# Supplementary material for: Photocatalytic Oxygen Evolution with Prussian Blue Coated ZnO Origami Core‐Shell Nanostructures
Source: Chemphyschem. 2025 Jan 29;26(6):e202400817. doi: 10.1002/cphc.202400817 (PMC11913467; doi:10.1002/cphc.202400817)
Supplement: Supplementary file 1 — Supporting Information [file CPHC-26-e202400817-s001.pdf]

ChemPhysChem

Supporting Information

Photocatalytic Oxygen Evolution with Prussian Blue Coated ZnO Origami Core-Shell Nanostructures

Ruby Phul, Guobin Jia, Emir Utku Sekercileroglu, Yves Carstensen, Ratnadip De, Andrea Dellith, Jan Dellith, Jonathan Plentz, Ferdi Karadaş,* and Benjamin Dietzek-Ivanšić*

Supporting Information

Photocatalytic Oxygen Evolution with Prussian Blue Coated ZnO Origami Core-Shell Nanostructures

Ruby Phul,[1,†] Guobin Jia,[2,†] Emir Utku Sekercileroglu,[1] Yves Carstensen,[3] Ratnadip De,[2,3] Andrea Dellith[2], Jan Dellith[2], Jonathan Plentz,[2] Ferdi Karadaş,[1,2,3,4 *] Benjamin Dietzek-Ivanšić[1,2,3 *]

1. Department of Chemistry, Bilkent University, 06800, Ankara, Türkiye

2. Leibniz Institute of Photonic Technology (Leibniz-IPHT), Albert-Einstein-Str. 9, 07745 Jena, Germany

3. Institute for Physical Chemistry and Abbe Center of Photonics, Friedrich Schiller University Jena, Helmholtzweg 4, 07743 Jena, Germany

4. UNAM – National Nanotechnology Research Center, Institute of Materials Science and Nanotechnology Bilkent University, 06800, Ankara, Türkiye.

† Contributed equally

*E-mail address: karadas@fen.bilkent.edu.tr and benjamin.dietzek@leibniz-ipht.de

Characterization:

X-ray diffraction studies were done within in the 2-theta range of 10-80° with a step size of 0.01 by using Panalytical X'pert Multi-Purpose X-ray diffractometer (MPD) with Cu Kα X-ray radiation (λ = 1.5418 Å). Scanning Electron Microscopy (SEM) images were obtained on a FEI-Quanta 200 FEG ESEM operated at 15 kV at Bilkent University as well as on a Field Emission Scanning Electron Microscopy JSM 6700F from JEOL at Leibniz Institute of Photonic Technology. A HT7820 (Hitachi) transmission electron microscopy (TEM) with an acceleration voltage of 120 kV was used to determine the shape and size of the samples, for which the powders were dispersed in ethanol, and a tiny droplet was drop casted on a TEM grid coated with a carbon film. X-ray photoelectron spectroscopy (XPS) measurement was performed on Thermo Scientific K-Alpha spectrometer, using Al K-Alpha radiation (hυ = 1486.6 eV), in survey mode. The binding energies were calibrated by referencing the C1s peak at 284.8 eV and shifting other peaks in the spectrum accordingly. Fourier transform infrared (FT-IR)

spectra were recorded with a Bruker Alpha Platinum-ATR spectrometer with 2 cm^{-1} resolution in a wavenumber range 4000-400 cm^{-1}.

Photocatalytic O_2 Evolution:

The photocatalytic experiments were carried out in a 15 mL round bottom flask at room temperature. In a typical experiment, the 5 mg of catalyst was dispersed in 10 ml of 5 mM aqueous solution of $Na_2S_2O_8$ (sacrificial agent/electron acceptor). The reaction solution was purged by bubbling N_2 gas and kept in the dark for 30 min to maintain the equilibrium with magnetic stirring. A 300 W Xe lamp (AM 1.5 global filter) was used as the light source. The amount of O_2 generated was determined at every 1 h intervals by injecting 100 µL of the reactor headspace gas into a gas chromatograph (Agilent 7820A GC, with molecular sieves column and a TCD detector). Ar was used as the carrier gas. Two rubber septa were used to seal the reactor, where the space between them was purged with N_2 gas to avoid a possible O_2 leak during the measurements. To check the stability of the catalysts, several photocatalytic experiments (18h) were conducted using the same reaction mixture, during each cycle the reaction mixture was centrifuged, and the pellet was washed with DI water and redispersed in 5 mM $Na_2S_2O_8$ solution and N_2 was bubbled into the solution for 30 mins.

Electrochemical Measurements:

The electrochemical measurements were done by a standard three-electrode Gamry Instruments Interface 1000 potentiostat/galvanostat, in a Bob's cell with Ag/AgCl (3.5 M KCl) as the reference electrode, a Pt wire as the counter electrode, and different ZnO samples coated FTO substrates as the working electrode. Before electrode preparation, the FTO substrates were cleaned by ultrasonication for 15 min each in soap solution, deionized water, acetone and isopropanol and then air dried at 100 °C for 1 h. The electrodes were prepared by drop-casting the 30 µl of catalyst ink (2 mg of ZnO or PB3@ZnO powder sonicated for 30 mins in 200 µl of IPA) on the 1x1 cm area of FTO substrate and dried at 60 °C for 10 min in an oven. All the experiments were performed in 0.1 M phosphate buffer solution (PBS; pH 7) containing 0.1 M KCl at room temperature. Before every experiment, the electrolyte was bubbled with N_2 gas for 15 min to remove dissolved O_2. Cyclic voltammetry (CV) measurements were conducted under dark conditions at a scan rate of 50 mV s^{-1} (Figure S1). The

Mott−Schottky analysis was carried out at 500 Hz frequency in dark and the flat band potentials were calculated using the following equation:

$$\frac{1}{C^2} = \frac{2}{\varepsilon_0 \varepsilon_r e N_D}\left[V - V_{FB} - \frac{k_B T}{e}\right]$$

where C represents the interfacial capacitance, ε_0 is the vacuum permittivity, ε_r is the dielectric constant of SC, N_D shows the carrier density, V is the applied voltage, k_B is the Boltzmann's constant, T represents the absolute temperature, and e stands for the electronic charge. The flat band potential (V_{FB}) was calculated by plotting the $1/C^2$ versus applied potential (V), represented by the potential at which the linear section crosses the horizontal axis.

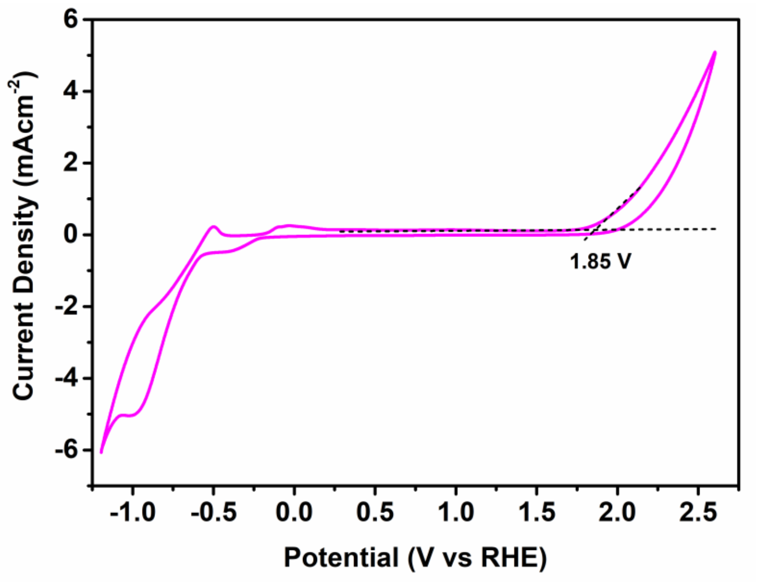

Figure S1. CV curve for PB3@ZnO in 0.1 M PBS electrolyte (pH 7) at the scan rate of 50 mV/s.

Post-catalytic Characterization.

To determine the changes that occurred in the PB3@ZnO photocatalyst during the photocatalytic experiments, post-characterization studies were carried out and compared with the pristine catalyst. The FT-IR spectra of the catalyst is shown in Figure S2, which indicates the cyanide stretching vibration of the catalyst was shifted slightly towards the lower wavelength after the long exposure to the solar illumination. Further, the post catalytic SEM analysis of ZnO and PB3@ZnO samples shows typical ultrathin nanosheets structure derived from the ZnO origami structure, and both samples are highly agglomerated either in the suspension or during the SEM sample preparation. (Figure S3).

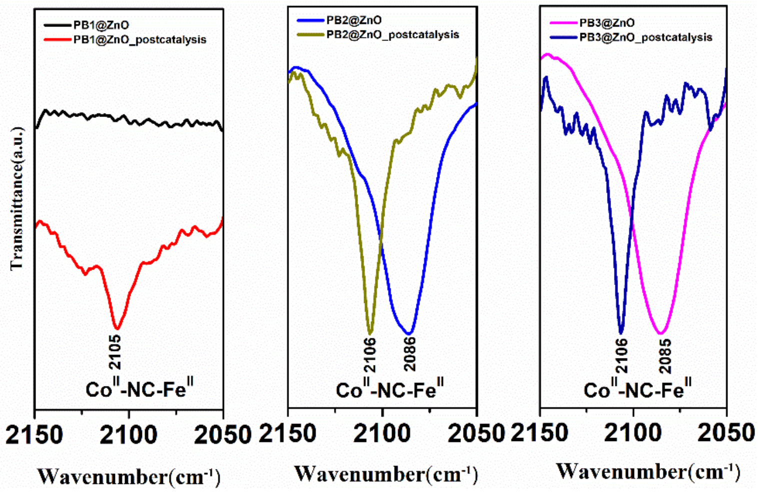

Figure S2. FT-IR spectra of pristine and postcatalysis FeCo@ZnO samples showing their cyanide stretching modes.

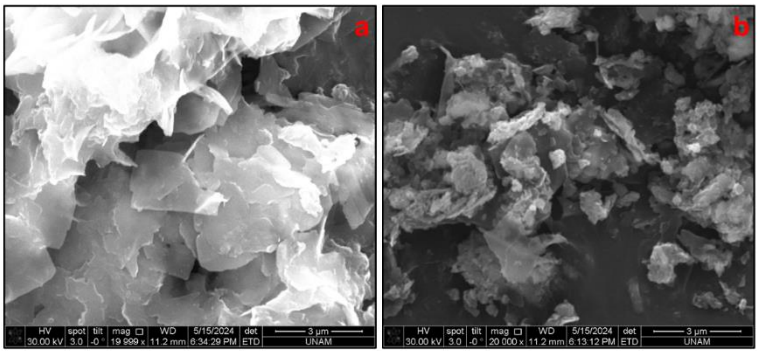

Figure S3. FE-SEM micrographs of postcatalytic (a) ZnO and (b) PB3@ZnO samples.
